# Does a colour-coded blood pressure diary improve blood pressure control for patients in general practice: The CoCo trial

**DOI:** 10.1186/1745-6215-11-38

**Published:** 2010-04-14

**Authors:** Claudia Steurer-Stey, Marco Zoller, Corinne Chmiel Moshinsky, Oliver Senn, Thomas Rosemann

**Affiliations:** 1 Institute for General Practice, University of Zurich, Sonneggstrasse 6, 8091 Zurich, Switzerland; 2 Department of Internal Medicine, University Hospital Zurich, Rämistrasse 100, 8091, Zurich, Switzerland

## Abstract

**Background:**

Insufficient blood pressure control is a frequent problem despite the existence of effective treatment. Insufficient adherence to self-monitoring as well as to therapy is a common reason. Blood pressure self-measurement at home (**H**ome **B**lood **P**ressure **M**easurement, **HBPM**) has positive effects on treatment adherence and is helpful in achieving the target blood pressure. Only a few studies have investigated whether adherence to HBPM can be improved through simple measures resulting also in better blood pressure control.

**Objective:**

Improvement of self-monitoring and improved blood pressure control by using a new colour-coded blood pressure diary.

**Outcome:**

Primary outcome: Change in systolic and/or diastolic blood pressure 6 months after using the new colour-coded blood pressure diary.

Secondary outcome: Adherence to blood pressure self-measurement (number of measurements/entries).

**Methods/Design:**

Randomised controlled study.

Population: 138 adult patients in primary care with uncontrolled hypertension despite therapy. The control group uses a conventional blood pressure diary; the intervention group uses the new colour-coded blood pressure diary (green, yellow, red according a traffic light system).

**Expected results/conclusion:**

The visual separation and entries in three colour-coded areas reflecting risk (green: blood pressure in the target range ≤ 140/≤ 90 mmHg, yellow: blood pressure >140/>90 mmHg, red: blood pressure in danger zone > 180 mmHg/>110 mmHg) lead to better self-monitoring compared with the conventional (non-colour-coded) blood pressure booklet. The colour-coded, visualised information supports improved perception (awareness and interpretation) of blood pressure and triggers correct behaviour, in the means of improved adherence to the recommended treatment as well as better communication between patients and doctors resulting in improved blood pressure control.

**Trial registration:**

ClinicalTrials.gov ID NCT01013467

## Background

Arterial hypertension is the most common cardiovascular risk factor throughout the world and guidelines for the management of hypertension underscore the need for strict blood pressure (BP) control to decrease the risk of morbidity and mortality in hypertensive patients [1,2]. Despite the available evidence that even mild hypertension is linked to a considerably higher cardiovascular risk, many patients do not reach the target blood pressure under treatment [3].

In Switzerland every seventh adult is affected, only half of them receive treatment. Of those patients treated, which cost the health system in Switzerland over CHF 435 million in 2005 [4], only one-eighth [5] reaches appropriate blood pressure control.

An important factor in achieving targets in the management of hypertension is patient adherence to treatment [6]. A plethora of data exists regarding adherence in the treatment of hypertension [7-12].

A review on how to reach targets in the treatment of diabetes patients in primary care showed that interventions with the greatest positive effect were complex and should include activities which change the behaviour of doctors, changes to practice organisation, expansion and provision of information and educational programmes for patients [13]. The measures to promote adherence in such programmes are the combination of patient information, promoting and supporting self-monitoring support through regular monitoring and inquiries by telephone, social support as well as treatment plans tailored to the individual [14]. Most of these measures require time and personal effort for training and follow-up, which is not easy to implement in the everyday practice life.

With a chronic illness as hypertension the role of the patient changes. The patient is central to efforts to monitor and control the illness to the best possible extent. Knowledge about illness and treatment is the basis, but in particular behaviour strategies are necessary to achieve the best possible long-term control. Our study focuses on patient-related factors as the central condition for improved treatment adherence [15].

Blood-pressure self-measurement at home (HBPM) is an established and evidence-based behaviour measure, which is recommended in guidelines on the treatment of hypertension and which has been shown to improve knowledge about treatment and also adherence to treatment with medication by about 20% [16-19]. Until now only few studies investigated the use of a simple practice tool in hypertensive patients to improve self-monitoring and adherence to HBPM considering its possible effect on blood pressure. We therefore aim to investigate the effect of a colour coded blood pressure diary following a traffic light system (green, yellow, red) on adherence to self-measurement and blood pressure monitoring.

### Hypothesis

The colour-coded blood pressure booklet improves adherence to self-monitoring (higher number of HBPM) and leads to improved blood pressure control.

We are assuming that the colour-coded tool supports adequate patient behaviours by improving awareness and interpretation of blood pressure control. It also might support better patient-doctor communication which is a central aspect for treatment adherence and improved blood pressure control.

## Methods/design

**Figure 1:**
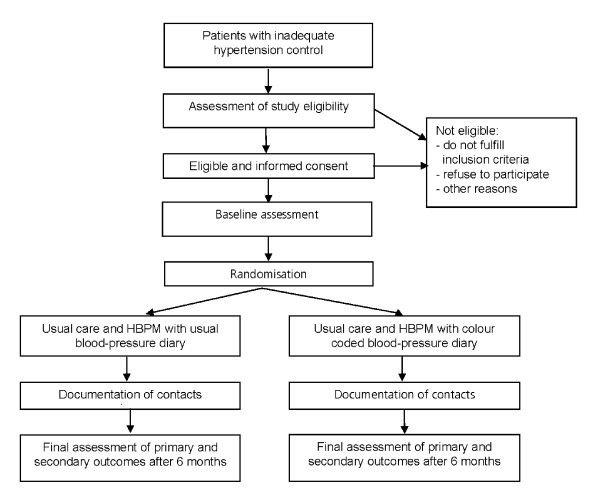
**Study flow chart**.

A two-armed study randomised and controlled at patient level. The intervention group will receive a new colour-coded blood pressure booklet, the control group a standard blood pressure record booklet without colour coding. The measuring period for both groups is 6 months (figure 1).

### Inclusion criteria for general practitioners

General practitioners will be invited to join the study via letters sent by post and information sessions. Any general practitioners who provide basic medial care with a workload of at least 20 hours per week are entitled to participate in the study.

### Inclusion criteria for patients

• Age >18 years

• BP > 140/90 mmHg

Two consecutive measurements carried out in the practice

(measured by the general practitioner or practice assistant at the start of the study; both measurements must be greater that 140/90 mmHg with the second measurement qualifying for inclusion.

• Unchanged anti-hypertension treatment for one month before inclusion

• Patient able to measure blood pressure at home

• Written informed consent

### Exclusion criteria

• Blood pressure reading over 180 mmHg systolic and/or 110 mmHg diastolic.

• Serious general or psychological illness (malignant tumours, serious depressive episodes or evidence of dementia).

• Insufficient knowledge of the German language for instruction and blood pressure recording with a booklet.

All patients will be asked consecutively about participating in the study and they will be handed an information sheet. All patients who have been approached will be included in a list containing the blood pressure booklet code which will be passed on to the study centre. The list remains in the practice so that the study leaders only have anonymous access to the patient data. However, in the practice it is possible to link the blood pressure record book with the medical file of the patient so that all the clinical data is available. Any patients who have been approached and have declined to participate will be included in a list in order to carry out a non-responder analysis of patients with regard to socio-demographic and clinical data.

#### Sample Size

Case number calculation and power analysis were carried out on the basis of the expected effect for systolic blood pressure. With an alpha of 0.05 and a power of 0.9 (one-sided test) this means that 138 patients are required in order to be able to show a clinically relevant blood pressure difference of 7 mm Hg systolic with a standard deviation of 14 mmHg between the groups. Assuming a dropout rate of 20% this means that at least 166 patients are required.

#### Randomisation

The randomisation (colour-coded vs. conventional blood pressure booklet) will take place at patient-level and will be carried out centrally by the study centre. We will draw up a randomising list by computer (ralloc command of Stata software for Windows, Version 9). The blood pressure record booklets will be distributed on the basis of this list in consecutively numbered opaque envelopes. The opaque envelopes will be given to the practices in numerical order. They can not be resealed. GPs are asked to notice the number (code) of the booklet (colour-coded or normal) on a list together with the name and birthday of the patient and generate a code out of this data. This list will remain in the practice and is just for the purpose to be able to link the booklets with the patients name and therefore the patients' medical file. The study centre will receive a list with the generated code of the patient and the distributed booklet. So, the linkage to the name and medical file is only possible with the list held by the GP. Opened but not used envelopes have to be noticed in the list as used ones and marked as "not distributed". The practices will each receive four envelopes in which there will either be a colour-coded or a standard record booklet. The patients will be included consecutively and the doctor will open one envelope after the other in the order of the envelope numbers. In practice number one envelopes 1-4 will be opened consecutively, in practice number two envelopes 5-8 etc. This central randomisation means that the concealment of random allocation is ensured.

### Blinding

Blinding of the general practitioners regarding group identity is not possible. Patients do only know that different types of blood pressure record booklets are being distributed, but they do not know in detail what the difference between the booklets is.

#### Intervention

The patients in the intervention group will receive a colour-coded blood pressure record booklet. The corresponding colour coded areas are divided into three areas of green, yellow and red following a traffic light system. The green area covers blood pressure readings up to a maximum of 140/90 mmHg, the yellow area includes systolic readings over 140 to 180 mmHg and diastolic readings over 90 to10 mmHg. The red area covers systolic readings over 180 mmHg and diastolic readings over 110 mmHg. All patients will be trained in the standardised use of the blood pressure record booklet including home blood pressure measurement additional they receive an instruction leaflet. They will be asked to measure their blood pressure in the morning while they are sitting and before they take their blood pressure medicaments. The readings will be recorded in the relevant blood pressure booklet for six months. All patients will be trained in the same way for one of the two study BP booklets by the general practitioner or the practice assistant for the duration of the study. The general practitioners and practice assistants will receive the same instruction and training for this as well as an instruction leaflet. Validated electronically blood pressure meters (MioStar Cardioplus 500) will be provided to all practices and patients to assure a standardization of the measurement. All measurements have to be performed with these meters.

### Data collection

#### Primary end point

There will be blood pressure measurements by the general practitioner at the start (t0) as well as after 3 months (t1) and at the end of the study after 6 months (t2). To rule out blood pressure differences BP will be measured at both arms at t0. Each subsequent measurement in the practice of the general practitioner will involve two blood pressure measurements in each case in the left arm if the initial measurement did not differ between both arms by more than 5 mm Hg. If the measurement differed by more than 5 mm Hg at t0, BP will be measured at the arm with the higher reading subsequently. All the readings will be recorded, but for inclusion (as stated above) as ell as for analysis, the second measurement will be used. The difference (t2-t0) between the systolic blood pressure readings at the practice measurement represents the primary end point.

All patient contacts will be collected over the entire period. At time t2 (after the end of the study) the number of times there was contact with the general practitioner since the inclusion of the patient will be recorded.

### Secondary end point

- Number of patients, who achieve blood pressure control under 140/90 mm Hg.

- Adherence: the absolute number of blood pressure measurements (entries in the BP record booklet) by the patients will be assessed as correlative of adherence during the period of observation.

The first supply of data takes place at randomisation and subsequent at the specific measuring points t1 and t2.

#### Analysis

At the end of the study the list with the booklet codes as well as the code, generated out of the patients' name and his birthday will be send to the study centre. In the study centre, all received questionnaires and lists will be scanned electronically and a TIF-file will be generated to avoid data manipulation. The data will be directly exported in the statistic program (STATA, version 9).

Descriptive statistics: average value with +/- 1 SD and median values of the average blood pressure readings for both groups. In accordance with the normal distribution test it will be decided whether parametric or non-parametric tests are to be applied. Treatment group comparisons for categorical data will be done with the χ^2 ^test. For continuous variables, comparisons between treatment groups were done with *t *tests.

Primary outcome: difference (t2-t0) between the systolic blood pressure average readings from the practice measurement. All patients will be analysed in accordance with the „intention-to-treat” approach and the latest available measurement will be used (last- observation carried forward). When interventions are provided via GPs, one should be aware of a cluster effect. We decided to perform a randomisation on the patient's level since the effect mediated by the GP on the intervention is assumed to be small. Nevertheless, we will account for that and include the factor "Practice" as a random effect in the analysis.

## Discussion

Although there is a large amount of data on non adherence in chronic illnesses such as diabetes, heart failure, chronic lung disease and hypertension, there is relatively little research data on interventions which try to solve the problem of non-adherence. In addition, the conclusions of intervention studies are often based on subjective patient information and not on objective investigations. Data and evidence on a true change in behaviour assessed with hard clinical end points are needed.

Our study measures the use of a simple, practicable intervention tool. A new BP record booklet containing and providing a visualised interpretation in colour of blood pressure control that is useful as feedback for the patient and doctor and can help to improve patient-doctor communication as well as triggering appropriate behaviour strategies. A change or adjustment in behaviours is an important precondition to achieve relevant clinical end points in the case of chronic illnesses (improvement of adherence, reaching treatment targets, reduction in complications and secondary failures as well as lowering direct and indirect costs).

If there is evidence of a significant effect with this simple intervention and the new hypertension control tool, then this benefit can be achieved without additional treatment and risk of side-effects, without relevant additional costs and without additional effort for the patient and doctor. Patients, who measure their blood pressure at home generally, record their blood pressure. The documentation in a different record booklet as used previously therefore means no extra effort. The instruction for using the new blood pressure record booklet is also no extra effort for the "health professionals", in this case the general practitioner or practice assistant. No extra work load is an important condition for implementation of evidence based recommendations into practice.

### Limitations of the study

A double blind approach is not possible because training has to be given on the blood pressure record booklet. Some sub-groups (age stratification, gender, education) are too small to reach significance.

#### Ethical principles

The study is being conducted in accordance with medical professional codex and the Helsinki Declaration as of 1996 as well as Data Security Laws.

Study participation of patients is voluntary and can be cancelled at any time without provision of reasons and without negative consequences for their future medical care.

#### Patient informed consent

Previous to study participation patients receive written and spoken information about the content and extent of the planned study; for instance about potential benefits for their health and potential risks. In case of acceptance they sign the informed consent form.

In case of study discontinuation all material will be destroyed or the patient will be asked if he/she accepts that existing material can be analyzed in the study.

#### Vote of the ethics committee

The study protocol is approved by the ethics committee of Zurich (Kantonale Ethikkommission (KEK) Zürich (EK-1738).

#### Data security/disclosure of original documents

The patient names and all other confidential information fall under medical confidentiality rules and are treated according to appropriate Federal Data Security Laws. All study related data and documents are stored on a protected central server of the University of Zurich. Only direct members of the internal study team can access the respective files.

Intermediate and final reports are stored at the Department of General Practice at the Zurich University Hospital (USZ).

## List of abbreviations

HBPM: Home Blood Pressure Measurement; BP: Blood Pressure; KEK: Kantonales Ethisches Kommitee.

## Competing interests

The authors declare that they have no competing interests.

## Authors' contributions

All five authors made a substantial contribution to this manuscript, i.e. conception, design and drafting.
